# AI-assisted human clinical reasoning in the ICU: beyond “to err is human”

**DOI:** 10.3389/frai.2024.1506676

**Published:** 2024-12-04

**Authors:** Khalil El Gharib, Bakr Jundi, David Furfaro, Raja-Elie E. Abdulnour

**Affiliations:** ^1^Division of Pulmonary and Critical Care Medicine, Rutgers Robert Wood Johnson Medical School, New Brunswick, NJ, United States; ^2^Division of Pulmonary and Critical Care Medicine, Brigham and Women’s Hospital and Harvard Medical School, Boston, MA, United States; ^3^Division of Pulmonary and Critical Care Medicine, Beth Israel Deaconess Medical Center and Harvard Medical School, Boston, MA, United States

**Keywords:** large language models, clinical reasoning, diagnostic errors, artificial intelligence, critical care

## Abstract

Diagnostic errors pose a significant public health challenge, affecting nearly 800,000 Americans annually, with even higher rates globally. In the ICU, these errors are particularly prevalent, leading to substantial morbidity and mortality. The clinical reasoning process aims to reduce diagnostic uncertainty and establish a plausible differential diagnosis but is often hindered by cognitive load, patient complexity, and clinician burnout. These factors contribute to cognitive biases that compromise diagnostic accuracy. Emerging technologies like large language models (LLMs) offer potential solutions to enhance clinical reasoning and improve diagnostic precision. In this perspective article, we explore the roles of LLMs, such as GPT-4, in addressing diagnostic challenges in critical care settings through a case study of a critically ill patient managed with LLM assistance.

## Introduction

Diagnostic error is a public health concern. It is estimated that nearly 800,000 Americans die or are permanently disabled by diagnostic error in various clinical settings each year (Newman-Toker et al., 2024). Globally, the incidence of diagnostic error is likely even higher as access to basic diagnostic testing resources can be limited in low-resource contexts, resulting in diagnostic delays for life-threatening diseases (Newman-Toker et al., 2024). Central goals of the initial clinical reasoning process are to reduce diagnostic uncertainty and communicate a plausible differential diagnosis for safe and effective patient care. However, the process frequently faces a variety of challenges including cognitive load, high patient complexity, and burnout leading to inefficiencies and diagnostic errors (National Academies Press, 2015). All these factors predispose clinicians to cognitive biases. Burnout may lead to an ‘availability bias’, wherein a clinician defaults to a familiar diagnosis rather than considering a broader range of possibilities, as it requires less mental effort. Addressing these challenges is crucial to mitigate reliance on heuristic shortcuts and improve diagnostic accuracy.

Critically ill patients are particularly prone to the harms from diagnostic errors, and by some estimates the prevalence of diagnostic error in patients admitted from the emergency department (ED) to the ICU exceeds 40% (Aaronson et al., 2020; Bergl et al., 2018). In addition, a meta-analysis demonstrated that ICU patients are twice as likely to have major misdiagnoses when compared to the patients admitted to the medical wards (Shojania et al., 2003). Moreover, it is estimated that up to 30% of patients with a diagnostic error in the ICU die secondary to this error (Auerbach et al., 2024). There is a critical need to uncover new approaches in clinical diagnosis and reasoning to improve patient outcomes, especially in the ICU. In this perspective article, we delve into the role of large language models (LLM) to address this important unmet clinical need as a framework to enhance the paradigm of human clinical reasoning in the ICU.

## Methods to improve diagnosis by enhancing clinical reasoning

The National Academy of Medicine describes improving diagnosis in healthcare as a “moral, professional, and public health imperative” (Singh and Graber, 2015). It articulated eight objectives to improve diagnosis, many of which target clinical reasoning, support clinical decision-making, and encourage cognitive forcing strategies and checklists (National Academies Press, 2015). Existing cognitive reasoning tools, such as reflection strategies or checklists result in clinically important improvements in diagnostic accuracy; however the overall impact is limited (Staal et al., 2022). In recent years, LLMs have been an emerging tool used in clinical settings with the goal of having a more meaningful effect (Lee et al., 2023). Although Artificial Intelligence (AI) tools have been used in healthcare for many decades, most have been trained on narrow datasets and provide support in specific contexts. In contrast, LLMs are generative AI tools trained on a vast text corpus. Therefore, by “hacking the operating system of human civilization” (Williams, n.d.), LLMs can provide support in many language-dependent domains, including medicine. In recent years, several LLMs have been developed, including BERT, XLNet, Pathways Language Model (PaLM), Open Pretrained Transformer (OPT), and the most globally used GPT (OSF, n.d.). The sheer number of parameters and the size of the training data of modern LLMs have opened many opportunities to support human cognitive tasks in the workplace (Thirunavukarasu et al., 2023). Currently, LLM applications are already being leveraged by clinicians as these new tools showed broad use cases, from drafting pre-authorization documents to transcribing and summarizing encounter notes (Lee et al., 2023; Locke et al., 2021). As such, LLMs have the potential to assist in many of the obstacles to diagnostic excellence in the ICU.

## Emerging diagnostic reasoning properties of LLM

Providing high-quality responses to medical questions requires an understanding of the medical context, recollection of pertinent knowledge, and human-like reasoning (Singhal et al., 2023). To validate the reasoning capabilities of LLMs in this domain, investigators tested them with licensing examinations (Singhal et al., 2023; Jin et al., 2020; Suchman et al., 2023). The results indicated that while their performance did not excel in certain assessments, they reached the requisite in others (Kung et al., 2023; Nori et al., 2023). Beyond answering test questions, LLMs have been evaluated for assessing patient scenarios and providing guidance about diagnosis and clinical reasoning (Liu et al., 2024). A recent study assessed the ability of LLMs’ to answer questions within critical care by extracting and responding to clinical concepts from the MIMIC III dataset, which contains medical information on patients admitted to critical care units (Liu et al., 2024). GPT-4 demonstrated superior performance compared to its predecessors, including GPT and LLaMA, providing answers that were relevant, clear, logical and more complete (Liu et al., 2024).

Research on LLMs’ diagnostic processes is advancing. When compared to human diagnosis, mixed results were seen with simple medical cases (Rao et al., 2023), complex cases (Kanjee et al., 2023) and gerontic ones (Shea et al., 2023), suggesting that with clinician-guided prompting and appropriate data input (Cabral et al., 2024), models could become more reliable. More recently, clinicians presented with challenging medical cases from the New England Journal of Medicine were compared in terms of their responses with and without LLM assistance. The LLM-assisted responses were more comprehensive and appropriate than those generated solely with textbooks and internet searches, highlighting the potential of these models as assistive tools in clinical decision-making (McDuff et al., 2023). When clinicians were provided with LLM support, their diagnostic accuracy improved, as these models could articulate clinical reasoning arguments assessed by validated rubrics. In situations where human clinicians and GPT-4 were given cases with unstructured data and asked to produce problem representations, clinical reasoning, and differential diagnoses, GPT-4 demonstrated better clinical reasoning with a similar level of diagnostic accuracy compared to humans (Cabral et al., 2024).

However, literature on LLMs’ capabilities in resolving critical care cases is limited. Critically ill patients often present with serious and complex multi-organ involvement, and require simultaneous diagnostic investigation and therapy, which makes the application of LLMs to these real-world situations fraught with difficulty. Herein, we present a case study of the diagnostic process of LLMs in a patient in the ICU at the Brigham and Women’s Hospital to assess their potential to enhance human diagnosis.

## Case study

**Figure 1:**
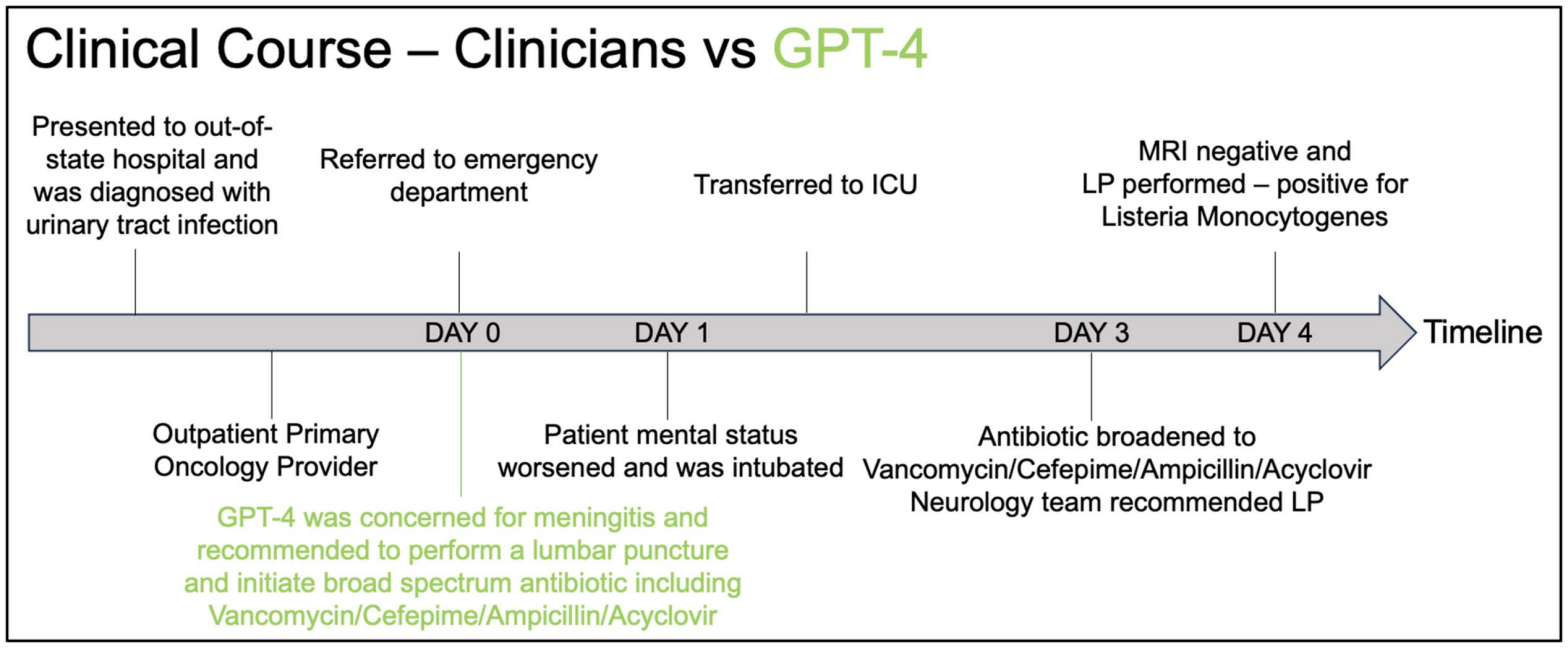
Timeline graphic comparing the clinical course of a patient as managed by clinicians (black) versus the recommendations made by GPT-4 (green).

A 61-year-old female with a history of hypertension, breast cancer status post bilateral mastectomy in 4 years prior to presentation, and recurrent ovarian cancer complicated by chemotherapy-induced thrombocytopenia, presenting with a five-day history of nausea, vomiting, diarrhea with poor oral intake, and three days of headache, altered mental status, confusion, slurred speech, and gait instability. The patient initially presented to an out-of-state hospital and was diagnosed with a urinary tract infection and was treated with intravenous fluid and piperacillin-tazobactam and was then sent home with a prescription for ciprofloxacin. The patient presented to her primary oncology provider the day after discharge where she was referred to the ED for further evaluation. The physical examination at the ED was notable for tachycardia, facial myoclonus, gait instability, and lower back tenderness. Labs demonstrated leukocytosis with a white blood cell of 36 K/uL, AST 148 U/L / ALT 81 U/L, ALP 328 U/L. Urinalysis revealed pyuria. Computed tomography (CT) of the head demonstrated no acute findings. The patient was admitted to the hospital. The next day, the patient had a fever with maximum temperature of 101.5F with worsening tachycardia and new oxygen requirement of 2 L nasal cannula to maintain oxygen saturation at >90%. The patient then developed worsening respiratory failure requiring emergent intubation shortly after undergoing computed tomography pulmonary angiogram for concerns for a pulmonary embolus. The patient was transferred to the ICU after intubation for further management. On hospital day 3, anti-microbials were broadened to Vancomycin/Cefepime/Ampicillin/Acyclovir per neurology recommendations to empirically treat meningitis while awaiting lumbar puncture. The next day, brain magnetic resonance imaging was negative for acute abnormality. Electroencephalogram revealed moderate bilateral cerebral dysfunction consistent with encephalopathy. Lumbar puncture was performed on hospital day 4 and yielded colonies of *Listeria monocytogenes* and blood cultures from two days prior also grew *Listeria monocytogenes*. A summary of the timeline of events is presented in Figure 1. More details of the events are presented in Supplementary material.

## Discussion

In Figure 1, we present the clinical course of the patient from the time they initially presented to the out-of-state hospital until the time of the final diagnosis. Using GPT-4, we provided a prompt (see Supplementary material) to instruct the model on providing a clinical summary with the top 10 differential diagnoses, additional diagnostic tests a physician should obtain, and initial management based on the history and physical written by the physician in the electronic medical record. The full response from GPT-4 is provided in Supplementary material. As indicated in red, the GPT-4 response recommended performing a lumbar puncture upon presentation at the ED and initiating broad-spectrum anti-microbials, including vancomycin, cefepime, ampicillin, and acyclovir, which was delayed by 48 h in the real-life scenario. This suggests a clinical utility of using LLM models in the care of critically ill patients to enhance our clinical reasoning and diagnostic processes, ultimately aiming to improve patient care.

**Figure 2:**
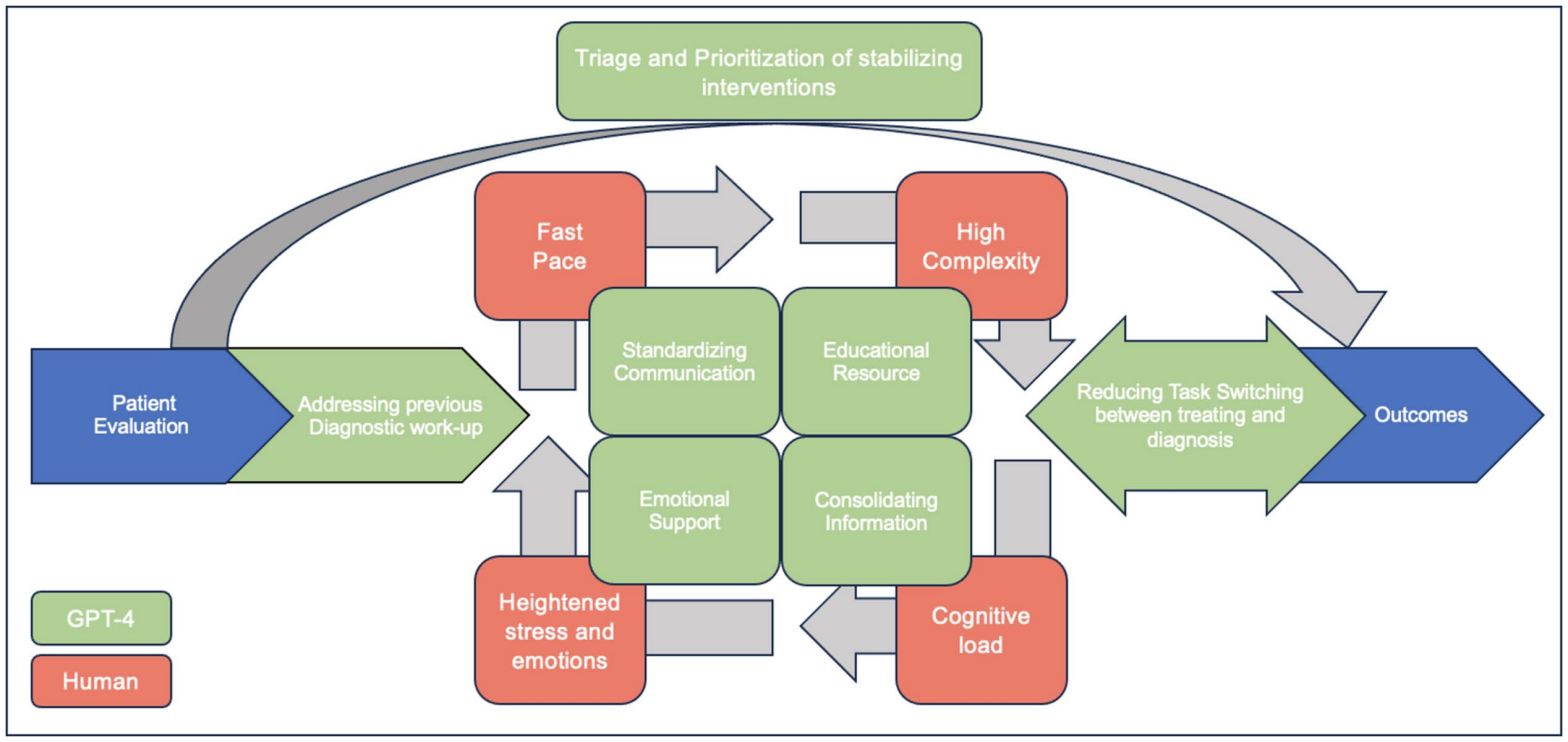
Enhancing ICU Patient Care with GPT-4: Addressing common hindrances to diagnosing critically ill patients (adapted with permission of the American Thoracic Society. Copyright © 2024 American Thoracic Society. All rights reserved (Bergl et al., 2018). Annals of the American Thoracic Society is an official journal of the American Thoracic Society. Readers are encouraged to read the entire article for the correct context at https://www.atsjournals.org/doi/10.1513/AnnalsATS.201801-068PS. The authors, editors, and The American Thoracic Society are not responsible for errors or omissions in adaptations).

In Figure 2, we highlight potential targets where LLMs can assist in the care of the critically ill. During the initial patient evaluation, physicians spend a significant amount of time reviewing patients’ previous diagnostic work-ups, which can sometimes be overwhelming. GPT-4 can aid in reviewing a patient’s history to identify potential diagnostic anchoring biases (McDuff et al., 2023). Additionally, LLMs could assist in triaging and prioritizing patients who need immediate intervention based on the acuity of their presentations. In addition, GPT-4 can help organize and summarize patient data, including history, lab results, and imaging, to provide clinicians with a concise overview (McDuff et al., 2023). GPT-4 can also provide differential diagnosis suggestions based on the presented symptoms and test results, helping to ensure that clinicians consider less common diagnoses they may have overlooked alongside the more common ones. In situations where complex management questions arise, GPT-4 can serve as an educational resource, providing quick access to relevant guidelines and literature (McDuff et al., 2023). While it cannot replace human compassion, GPT-4 can offer support to healthcare workers under stress by providing a space to quickly debrief or reflect on difficult cases, which may help manage the emotional toll of healthcare work (Tu et al., 2024).

While the proof-of-concept case presented here demonstrates the potential benefits of LLMs, we acknowledge the limitations inherent in using a single case study to justify the broader application of LLMs in clinical reasoning. It is essential to approach the use of LLMs with caution, recognizing their limitations and potential biases. LLMs are trained on extensive datasets that may include biased information, leading to skewed responses. Additionally, the complexity of medical decision-making, characterized by nuanced and context-specific knowledge, can be challenging for LLMs, which rely heavily on pattern recognition rather than deep understanding. LLMs can also produce hallucinations, generating plausible-sounding but incorrect information, which can be dangerous in a clinical setting. Moreover, ethical and legal implications must be carefully considered, including potential malpractice issues and the necessity of informed consent for patients. Developing robust regulatory frameworks will be crucial to responsibly harness the potential of LLMs in clinical practice. Further research is needed to evaluate the effectiveness and safety of LLMs in supporting ICU clinical reasoning.

## Conclusion

Humans err, and errors are expensive and harmful in the healthcare setting. Diagnostic error remains a hidden epidemic in the ICU. It is time for the critical care community to acknowledge the gravity of the issue and recognize the potential for emerging technologies like LLMs to serve as pivotal allies in the ICU. The proof-of-concept case study presented here, along with the proposed integration of GPT-4 into the clinical workflow, has demonstrated the potential advantages and enhancements to diagnostic accuracy that LLMs can offer. As we find ourselves on the brink of a new era in medicine, it is becoming increasingly clear that the judicious use of AI, exemplified by LLMs, can usher in a paradigm shift toward more precise, efficient, and compassionate care in the ICU. To fully realize this potential, it is essential to educate physicians in the use of LLMs to augment their diagnostic and clinical reasoning skills. With ongoing research, refinement, and integration, LLMs could well become an indispensable component of critical care, mitigating the risk of diagnostic errors and elevating the standard of patient care to unprecedented heights.

## Data Availability

The datasets presented in this study can be found in online repositories. The names of the repository/repositories and accession number(s) can be found in the article/Supplementary material.
