# Supplementary material for: AI-assisted human clinical reasoning in the ICU: beyond “to err is human”
Source: Front Artif Intell. 2024 Dec 4;7:1506676. doi: 10.3389/frai.2024.1506676 (PMC11659639; doi:10.3389/frai.2024.1506676)
Supplement: Supplementary file 1 [file Data_Sheet_1.DOCX]

Supplementary Data 1.

GPT-4 PROMPT

I am a pulmonary and critical care physician taking care of patients in the medical intensive care unit (ICU) in an academic hospital in Boston. My main role is to evaluate patients for the possible need for ICU level of care in the emergency department (ED). Before I see the patient, I will get a consult from the emergency medicine physician regarding a sick patient in the ED. I will review their chart including their past medical history, labs, and imaging, interview and examine patient at bedside, and build a problem list with differential diagnosis with a management plan. I want you to be the consultant on the team. I will provide you the history of present illness with the vitals, physical examination findings, labs, and imaging data. Following that, I want you to provide me with 1) a brief summary of the patient, 2) 10 Differential diagnosis (ranked by percentage likelihood), 3) further diagnostic tests you would obtain and 4) an initial management plan.

LINK TO CHAT ON GPT-4:

https://chat.openai.com/share/6f49bd8e-db76-431d-a62d-3310147803f1

INPUT

Patient is a 61 y.o. female with a history of HTN, breast cancer s/p bilateral mastectomy (2009), and recurrent ovarian cancer complicated by chemotherapy-induced thrombocytopenia, presenting with 5 days of nausea with poor PO intake, and 3 days of altered mental status. History was mostly provided by patient's daughter at the bedside. Current illness began 5 days ago and developed nausea with vomiting. She didn't have her antiemetics with her, so she was unable to eat anything all weekend, though was drinking ~8 cups of water daily. She returned home 2 days ago, and at that time a friend noticed she wasn't safe to drive as she seemed a bit off, so escorted her home. Yesterday her daughter spoke with her by phone and noted that she had slurred speech, so she picked her up from work. She noted that her mom was disheveled with unbrushed hair and not in her typical work clothes. At home she slept from 1-7pm and woke up more confused. So last night they took her to an out-of-state hospital.

She was diagnosed with a UTI and treated with IVF and Zosyn and sent home with a script for

Ciprofloxacin. Per report, they recommended scans, but patient declined and went home overnight, preferring to seek care at the primary oncologist clinic. Today her daughter reports she is mentally better than yesterday, but still not back to herself. Speech is slower than usual, and she is using the walls for support when walking down the hallway. Daughter endorsed that her mother has a generalized headache (4/10 on the pain scale) both yesterday and today, similar to priors that she gets once every 2 weeks, for which she has taken acetaminophen. Denied fevers, chills, night sweats, dizziness, lightheadedness, unilateral weakness, vision changes, photophobia, dysuria, flank pain, shortness of breath, cough, chest pain, numbness/tingling. Endorsed abdominal pain similar to baseline. Endorsed nausea, but no emesis in recent days. Endorsed low back pain. Endorsed constipation at baseline, with

small bowel movement yesterday and 2 days ago. Physical examination: GENERAL: Alert female, in no acute distress, non-toxic appearing. HEAD: Atraumatic, normocephalic. EYES: PERRL, EOMI bilaterally. No injection, drainage or periorbital edema. No nystagmus or photophobia. NOSE: Nasal passages clear without drainage MOUTH: Mucous membranes moist, oropharynx without erythema, exudate or tonsillar enlargement. Airway widely patent. No mucosal lesions. NECK: Supple, no tenderness, no meningism. CV: RRR, no murmurs, rubs or gallops. PULM: Lungs clear to auscultation bilaterally. No wheezes, rhonchi or rales. ABD: Active bowel sounds. Soft, tender with deep palpation in RUQ/epigastric/LUQ/LLQ. Nontender in RLQ. BACK: No soft tissue swelling, tenderness. No focal thoracic or lumbar spinal tenderness. No CVA tenderness bilaterally. EXT: No edema. +DP pulses bilaterally NEURO: Patient alert and oriented x 4, clear but slowed/halted speech, slow gait with hands

CN 2-12 intact. No pronator drift, tongue midline. Extremities with equal 5/5 strength

bilaterally. MSK: No focal joint swelling or tenderness. Full ROM all 4 extremities. SKIN: No rash,erythema, tenderness, swelling. Labs: • APTT 46.6 (H) • PT 14.4 • INR 1.1 • FIBRINOGEN 249 • WBC 20.46 (H) • RBC 4.27 • HGB 14.0 • HCT 42.1 • PLT 22 (L) • MCV 98.6 (H) • MCH 32.9 • MCHC 33.4 • RDW 15.7 • MPV 9.5 • NRBC 0.00 • ABSOLUTE NRBC Not Reported • BLASTS 0.0 0 % • NEUTS (MANUAL) 93.0 (H) • EOSINOPHIL 0.0 (L) • BASOPHIL 0.0 (L) • LYMPHS 4.7 (L) • MONOS 2.3 (L) • ABSOLUTE BLASTS 0.00 • ABSOLUTE NEUTS 9.03 (H) • ABSOLUTE EOS 0.00 • ABSOLUTE BASO 0.00 • ABSOLUTE LYMPHS 0.96 • ABSOLUTE MONOS 0.47 • PLATELET ESTIMATE DECREASED • ANISO SLIGHT • OVALOCYTES FEW • POIKILOCYTOSIS SLIGHT • POLYCHROMESLIGHT • TOXIC GRANULATION PRESENT • SODIUM 129 (L) • POTASSIUM 3.4 • CHLORIDE 91 (L) • CO2 25 • BUN 13 • CREATININE 0.68 • GLUCOSE 98 • ALBUMIN 3.5 • TOTAL PROTEIN 6.0 (L) • CALCIUM 8.7 (L) • ALKALINE PHOSPHATASE 328 (H) • TOTAL BILIRUBIN 0.9 • AST 148 (H) • ALT 81 (H) • GLOBULIN 2.5 • EGFR 99 • ANION GAP 13 • MAGNESIUM 1.4 (L) • COLOR Yellow •CLARITY Clear • GLUCOSE NORMAL • BILI Negative • KETONES 1+ (*) ACETEST NEGATIVE •

SPECIFIC GRAVITY 1.027 • BLOOD 2+ (*) • PH 5.5 • Protein-UA 1+ (*) • UROBILINOGEN 1+ (*) • NITRITE Negative • Leukocyte esterase, urine Positive (*) • WBC 17 (H) • RBC 3 (H) • BACTERIA None • SQUAMOUS CELLS <1 (*) None /hpf • MUCUS Trace (*) • LIPASE 87 (H) CT HEAD WITHOUT CONTRAST TECHNIQUE: Multidetector-row CT of the head was performed without intravenous contrast using tailored dose modulation techniques. Images were reconstructed in the axial, coronal, and sagittal planes. COMPARISON: None FINDINGS: Brain Parenchyma: No midline shift, mass effect, parenchymal hemorrhage, or evidence of acute territorial infarct. There are areas of hypodensity in the periventricular white matter, likely a manifestation of chronic small vessel disease. Ventricular System and Extra-Axial Spaces: No extra-axial fluid collections. Basal cisterns are patent. No hydrocephalus. Partially empty sella. Osseous and Extracranial Structures: No significant paranasal sinus disease. No orbital abnormality. Right temporomandibular degenerative changes. Impression: No acute intracranial findings.

ICU Consultant

Summary of Patient Data: The patient is a 61-year-old female with a history of hypertension, breast cancer, and recurrent ovarian cancer, presenting with a recent history of nausea, poor oral intake, and altered mental status following a ferry ride. She was recently diagnosed with a UTI and treated with IV fluids and Zosyn. The patient shows signs of confusion, slurred speech, and requires support while walking. She has generalized headaches, abdominal pain, nausea, and constipation. Lab results indicate thrombocytopenia, elevated WBC count, altered liver enzymes, and signs of a urinary tract infection. CT head shows chronic small vessel disease but no acute findings.

Differential Diagnosis (with estimated likelihoods):

1. Metabolic Encephalopathy (30%): Altered mental status could be due to electrolyte imbalances (hyponatremia) and hepatic dysfunction indicated by abnormal liver enzymes.

2. Sepsis/UTI (20%): High WBC count and symptoms of UTI, alongside altered mental status,

suggest possible sepsis.

3. Cerebral Metastasis (10%): History of cancer raises the possibility of metastases, although not seen on the CT scan.

4. Chemotherapy-Induced Neurotoxicity (10%): History of chemotherapy could contribute to

altered mental status.

5. Dehydration (10%): Poor oral intake and ongoing nausea could lead to dehydration, affecting

mental status.

6. Stroke (5%): Although the CT scan is unremarkable, a history of cancer and thrombocytopenia increases the risk.

7. Hepatic Encephalopathy (5%): Altered liver enzymes could suggest hepatic dysfunction.

8. Medication Side Effects (5%): Reaction to new or existing medications cannot be ruled out.

9. Chronic Subdural Hematoma (2.5%): In the context of thrombocytopenia, even minor trauma

could lead to hematoma, but not evident on CT.

10.Thiamine Deficiency/Wernicke's Encephalopathy (2.5%): Poor nutritional intake raises this

possibility.

Testing and Treatment Plan:

1. Testing:

• Repeat electrolytes, including magnesium and calcium.

• Liver function tests to further assess hepatic status.

• MRI brain to rule out cerebral metastasis or other pathologies not visible on CT.

• Blood cultures and further urine analysis to confirm UTI and assess for sepsis.

• ECG and cardiac enzymes to rule out myocardial infarction, which can present atypically.

2. Treatment:

• IV fluids to address dehydration and electrolyte imbalances.

• Broad-spectrum antibiotics for UTI and possible sepsis, adjusted based on culture results.

• Thiamine supplementation given poor nutrition.

• Review and possibly adjust current medications.

• Supportive care for nausea and constipation.

• Monitor platelet count and manage thrombocytopenia appropriately.

• Regular neurologic assessments to monitor mental status changes.

Close monitoring and reassessment are vital, given the complexity of the patient's presentation and medical history. Further adjustments in the management plan should be based on the results of the additional tests and the patient's response to the initial treatments.

INPUT

A few days later, patient remains to be symptomatic and was transferred from the outpatient oncologist and was referred to the emergency department. Here is the data below from patient chart. Any additional thoughts to what you just explained: Patient a 61 year old. female with a history of hypertension, breast cancer s/p bilateral mastectomy (2009), and recurrent ovarian cancer complicated by chemotherapy-induced thrombocytopenia, presenting with 5 days of nausea with poor oral intake, and 3 days of altered mental status and gait instability. Pt reports that she came to the hospital from an oncology clinic appointment at the encouragement of her daughter because she has not been "herself" lately. Specifically, over the past three days, patient has noticed that she has been more confused than normal, speaking more slowly and with more slurred speech, and intermittently sending incomprehensible texts, along with forgetting normal daily activities such as brushing her hair. This confusion has been somewhat waxing and waning and, at the time of the interview, patient reports that she is at least close to her baseline mentation. For context, patient works full-time in an academic setting at a nearby university. She has noticed more difficulty focusing over the last few weeks, though her confusion distinctly worsened in just the past few days. Additionally, patient has had increased gait instability over the past three days. She notes that she lives alone and typically is able to walk around without issue, but has had to hold onto railings and use the wall for support to keep from falling. Denies any falls, loss of consciousness, or presyncope. Patient also reports decreased oral intake over the past ~1 week, only eating a few bites of food over the past several days and with relatively minimal fluid intake. Also having headache and lightheadedness over the past few days. Patient states that she had one episode of small volume emesis which was clear. She notes that she has mild, lower abdominal pain and increased urinary urgency over the last few days. She also has Right>Left lower back pain.

She denies dysuria, fevers, chills, or noticeable change in urine color or smell. Regarding her headache, patient reports that it feels like pressure across her forehead and is comparable to headaches she has previously had. States that she regularly gets headaches. At this time, she describes her headache as a 8/10 pain. Can walk up a few flights of stairs. Works in engineering at a college. Last two weeks, it has been harder to focus. Loose bowel movement a few days ago with most recent bowel movement. Denies chest pain, shortness of breath, difficulty breathing, no abdominal pain. Does not feel lightheaded when she stands. No incontinence. Current Vitals: 36.8 °C (98.2 °F) Pulse 103 Blood Pressure 139/78 RR 32 SpO2 95% at room air Weight 59.3 kg Body mass index is 23.91 kg/m².

Physical Exam

Constitutional: General: She is not in acute distress. Appearance: Normal appearance.

HENT: Head: Normocephalic and atraumatic. Right Ear: External ear normal. Left Ear: External ear normal. Nose: Nose normal. Mouth/Throat: Mouth: Mucous membranes are moist. Comments:

Repetitive movements of mouth and scrunching of nose that pt reports is not normal for her Eyes: General: No scleral icterus. Right eye: No discharge. Left eye: No discharge. Cardiovascular: Rate and Rhythm: Regular rhythm. Tachycardia present. Pulses: Normal pulses. Heart sounds: No murmur heard. No friction rub. No gallop. Pulmonary: Effort: Pulmonary effort is normal. No respiratory distress. Breath sounds: No wheezing, rhonchi or rales. Abdominal: General: Abdomen is flat. Palpations: Abdomen is soft. Tenderness: There is right CVA tenderness. There is no left CVA tenderness, guarding or rebound. Comments: Mild suprapubic tenderness Musculoskeletal: General: No swelling or tenderness. Cervical back: Normal range of motion. No rigidity. Comments: Trace BLE edema Skin: General: Skin is warm and dry. Capillary Refill: Capillary refill takes less than 2 seconds. Comments:

Diffusely erythematous Neurological: Mental Status: She is alert and oriented to person, place, and time. Comments: CN II-XII intact, 5/5 strength in bilateral upper and lower extremities, intact sensation to light touch, no increased muscle tone, normal Romberg, no asterexis or clonus Psychiatric: Mood and Affect: Mood normal. Behavior: Behavior normal. Labs: WBC 20.46 Hb 14 Platelet 22 SODIUM 129* POTASSIUM 3.4 CHLORIDE 91* CARBON DIOXIDE 25 BUN 13 CREATININE 0.68 ANION GAP 13 GLUCOSE 98 CALCIUM 8.7* MAGNESIUM 1.4* PHOSPHORUS -- Coags Recent Labs PT 14.4 PTT 46.6* PT-INR 1.1 Micro: Reviewed Microbiology Results (Last 10 days) ** No results found for the last 240 hours. ** Studies: Reviewed CT Head - No acute intracranial findings.

ICU Consultant

Updated Assessment:

The patient, a 61-year-old female with a complex medical history, continues to exhibit symptoms of altered mental status, gait instability, decreased oral intake, and now presents with higher intensity headaches and respiratory rate. She has a history of hypertension, breast cancer, recurrent ovarian cancer, and thrombocytopenia. Recent lab findings show elevated WBC, thrombocytopenia, and mild electrolyte imbalances. The CT head scan remains unremarkable for acute intracranial findings.

Revised Differential Diagnosis:

1. Cerebral Metastasis (15%): The worsening of cognitive and neurological symptoms could

indicate metastasis, despite not being evident on CT.

2. Metabolic Encephalopathy (15%): Persistent electrolyte imbalances and renal function

abnormalities may contribute to altered mental status.

3. Chemotherapy-Induced Neurotoxicity (10%): Ongoing chemotherapy may cause neurotoxic

effects, worsening cognitive function.

4. UTI and Possible Sepsis (10%): High WBC and symptoms suggest an ongoing infection,

potentially leading to sepsis.

5. Meningitis/Encephalitis (10%): CNS infection should be considered, especially given the

patient's immunocompromised state.

6. Hepatic Encephalopathy (10%): Altered liver function tests indicate potential hepatic

involvement.

7. Stroke (10%): Although not evident on CT, the patient's history and symptoms warrant

consideration.

8. Dehydration and Malnutrition (10%): Limited intake may exacerbate her symptoms.

9. Medication Side Effects (5%): New or existing medications could be contributing to her

symptoms.

10.Psychiatric Illness (5%): Psychiatric disorders or stress-related cognitive impairment could be

considered.

Revised Testing and Treatment Plan:

1. Testing:

• Repeat full metabolic panel, liver function tests, and complete blood count.

• Lumbar puncture to evaluate for CNS infection.

• MRI of the brain to rule out cerebral metastasis or other pathologies.

• Echocardiogram to assess cardiac function, considering tachycardia and increased

respiratory rate.

• Urine culture to confirm UTI and assess for other pathogens.

2. Treatment:

• Continued IV fluids for hydration and electrolyte correction.

• Review and possibly revise antibiotic therapy based on new culture results and sensitivity.

• Nutritional support, considering her poor oral intake.

• Pain management for her headaches.

• Thiamine and multivitamin supplementation.

• Close monitoring of neurological status and vital signs.

It's essential to continue multidisciplinary care, involving oncology, neurology, and possibly infectious disease specialists, given the complexity of her case. The treatment plan should be dynamically adjusted based on her response and new test results.

INPUT

Based on this data, what choice of anti-microbial would you consider in this patient suspecting the sources of infection indicated here?

ICU Consultant

Given the patient's clinical presentation and the potential sources of infection, the choice of

antimicrobials should cover the most likely pathogens while considering her history of recent antibiotic use, allergies, and the potential for drug interactions. Based on the provided information, the following antimicrobial choices can be considered:

1. For Urinary Tract Infection (UTI):

• Empiric Therapy: A third-generation cephalosporin like ceftriaxone or a fluoroquinolone

like levofloxacin could be considered. However, the recent use of Zosyn (piperacillin/tazobactam) and Cipro (ciprofloxacin) needs to be taken into account, as

resistance to these drugs might have developed.

• Culture-Directed Therapy: If urine cultures and sensitivities are available, antibiotic choice should be adjusted accordingly.

2. For Possible Central Nervous System (CNS) Infection:

• Empiric Therapy: In the absence of lumbar puncture results, broad coverage including

vancomycin (for gram-positive coverage, including resistant strains like MRSA), ceftriaxone or cefepime (for gram-negative coverage), and ampicillin (for Listeria coverage, particularly important in immunocompromised patients) should be considered.

• Adding Acyclovir: If viral encephalitis is a concern, acyclovir should be started empirically.

3. For Sepsis:

• Broad-Spectrum Coverage: Given the high WBC count and the possibility of sepsis,

broad-spectrum antibiotics like meropenem or imipenem-cilastatin could be considered,

especially if there's a concern for resistant organisms or polymicrobial infection.

4. Special Considerations:

• Renal Function: The patient's renal function should be monitored, and dosages adjusted accordingly.

• Antifungal Therapy: If there's a suspicion of a fungal infection (less likely but possible in

immunocompromised patients), antifungal therapy like fluconazole or an echinocandin (e.g., caspofungin) may be warranted.

• History of Cancer and Chemotherapy: This history increases the risk for opportunistic

infections, so a broader range of pathogens may need to be covered. It's crucial to monitor the patient's response to therapy and to adjust the antimicrobial regimen based on culture results, clinical improvement, and potential side effects. Additionally, consulting with an infectious disease specialist could be beneficial in managing such a complex case.
